# **gmXtal**: Cooking Crystals with GROMACS

**DOI:** 10.1007/s10930-023-10141-5

**Published:** 2023-08-25

**Authors:** Pavel Buslaev, Gerrit Groenhof

**Affiliations:** Department of Chemistry and Nanoscience Center, University of Jyväskylä, 40014 Jyväskylä, Finland

**Keywords:** GROMACS, Molecular dynamics, Crystal, Protein

## Abstract

Molecular dynamics (MD) simulations are routinely performed of biomolecules in solution, because this is their native environment. However, the structures used in such simulations are often obtained with X-ray crystallography, which provides the atomic coordinates of the biomolecule in a crystal environment. With the advent of free electron lasers and time-resolved techniques, X-ray crystallography can now also access metastable states that are intermediates in a biochemical process. Such experiments provide additional data, which can be used, for example, to optimize MD force fields. Doing so requires that the simulation of the biomolecule is also performed in the crystal environment. However, in contrast to simulations of biomolecules in solution, setting up a crystal is challenging. In particular, because not all solvent molecules are resolved in X-ray crystallography, adding a suitable number of solvent molecules, such that the properties of the crystallographic unit cell are preserved in the simulation, can be difficult and typically is a trial-and-error based procedure requiring manual interventions. Such interventions preclude high throughput applications. To overcome this bottleneck, we introduce **gmXtal**, a tool for setting up crystal simulations for MD simulations with GROMACS. With the information from the protein data bank (rcsb.org) **gmXtal** automatically (i) builds the crystallographic unit cell; (ii) sets the protonation of titratable residues; (iii) builds missing residues that were not resolved experimentally; and (iv) adds an appropriate number of solvent molecules to the system. **gmXtal** is available as a standalone tool https://gitlab.com/pbuslaev/gmxtal.

**Graphical Abstract:**

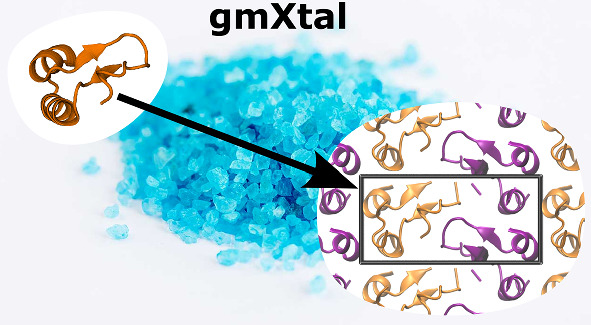

## Introduction

Multi-dimensional Nuclear Magnetic Resonance (NMR) spectroscopy can provide structural and dynamical information on biomolecules in solution, but this technique is limited to macromolecules of up to 35 kDa [1]. In contrast, in X-ray crystallography there is no such limit [2, 3], but this technique can only provide static information about protein structure in a crystal environment, where there are strong inter-molecular interactions between the proteins. With the development of free electron lasers and time-resolved techniques, also protein dynamics can now be probed by X-ray crystallography, but the limitation of the non-native crystal environment remains [4].

Protein dynamics in solution have therefore been predominantly investigated by means of molecular dynamics (MD) computer simulations [5]. The initial structure for such simulations is normally prepared by placing a single asymmetric unit from an X-ray crystal structure at the center of a periodic simulation box that is subsequently filled with water molecules, ions and other constituents if needed. However, because the protein environment in the simulations differs significantly from that in the crystallography experiment, comparing observables obtained from MD simulations with observables from crystallography remains challenging. Already in the early days of MD, Herman Berendsen et al. had addressed this issue and compared trajectories of proteins in solution and crystal environments [6, 7]. Later, it was demonstrated that proteins simulated in solution can relax into a conformation that is different from the X-ray structure [8–10]. Thus, to validate MD simulations in general and force fields in particular, with either static or dynamic X-ray data, simulations would need to be performed of protein in crystals, rather than solution [11–20].

To routinely perform MD simulations of protein crystals, efficient standardized approaches are needed for building crystal models. Whereas for setting up simulations of proteins in solution, a wide range of user-friendly methods has been introduced [21–23], the number of tools available to create a crystal model is much more limited. Although good guidelines exists, for example the tutorial of Cerutti and Case [24], and also CHARMM-GUI can generate protein crystals [21], these approaches are difficult to scale up for high-throughput applications, which would be needed for a systematic validation and improvement of force fields based on X-ray data. Furthermore, because not all solvent molecules are typically resolved in X-ray structures, determining how many solvent molecules are needed to preserve the volume of the unit cell while sampling the isothermal-isobaric ensemble in MD simulations under periodic boundary conditions, remains a major challenge that is currently not resolved. Instead, the addition of solvent molecules, which is essential to preserve the volume of the unit-cell, requires manual intervention, often by trial and error.

To overcome these challenges and provide the community with an efficient tool for setting up protein crystals for MD simulations in an automated and hence reproducible manner, we have implemented **gmXtal**, a combination of python scripts that automatically constructs the unit-cell, estimates the correct number of solvent molecules to be added and prepares the workflow for running MD simulations of the hydrated crystal with the GROMACS MD program [25, 26]. This paper is organized as follows: we first introduce the steps of the **gmXtal** workflow, we then provide the technical details of the simulations we performed, and finally demonstrate how **gmXtal** works by building crystals of three representative biomolecules.

## Methods

### gmXtal

**Fig. 1:**
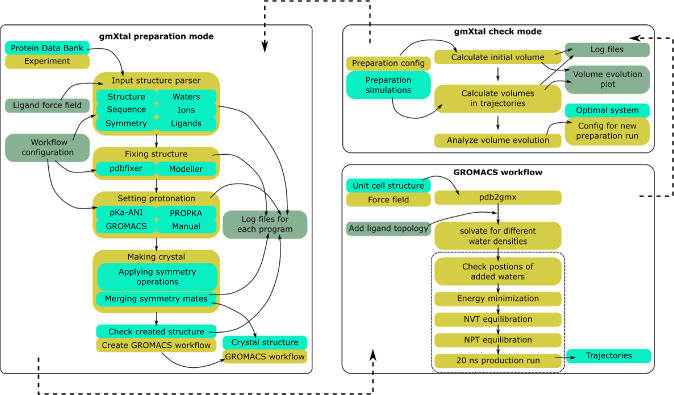
**gmXtal** consists of three main parts: (i) a preparation module, (ii) a GROMACS [25, 26] workflow, and (iii) a check module. The preparation module retrieves the structure from the protein data bank [27] or uses a structure provided by the user, cleans the structure, builds the crystal and creates a bash script that sets up a GROMACS workflow. The GROMACS workflow solvates the crystal structure at different water concentrations, runs minimization, NVT and NPT equilibrations, and 20 nanosecond production runs. The check module analyses the evolution of the simulation box volume for all production runs and compares the volume of systems with different numbers of added water molecules to the unit cell volume from the X-ray structure file. If none of the simulated systems preserves the experimental volume, the check module will suggest new parameters for the preparation module. Otherwise, it will recommend using the system that preserves the volume, for further production runs

**gmXtal** is a python-based toolbox, which automatically builds structures, generates topologies, and prepares scripts for simulating biomolecular crystals with GROMACS [25, 26]. **gmXtal** consists of three modules: (i) a preparation module, (ii) a GROMACS workflow, and (iii) a check module (Fig. 1), which are explained in detail below. **gmXtal** can also be used for setting up simulations of biomolecules in solution, if requested. Building crystals of membrane proteins is currently not supported.

#### Preparation Module

The preparation module cleans the structure, builds the crystal and prepares a bash script for running a GROMACS workflow. The structure can either be downloaded from the protein data bank [27], or provided by the user. **gmXtal** works with PDBx/mmCIF format [28], which is currently the standard file format of the protein data bank. Structure cleaning includes (i) selecting alternative conformations and preferred conformers of redidues, which are decided by the user, (ii) fixing missing residues based on the sequence information, and (iii) determining the optimal protonation state of titratable amino acids at the pH value selected by the user. Addition of missing residues is required to complete protein chains. Residues that are missing in the experimental structures, are from parts of the biomolecule that are less structured and hence do not give rise to Bragg peaks. Therefore, the resulting model structure might deviate from the real conformation, which can lead to bias in the simulation. Because adding missing residues can furthermore introduce steric clashes, **gmXtal** checks for such clashes and notifies the user. Structure cleaning also takes care of crystal waters, ions, and ligands, if a user wants these to remain included in the simulation box. Note, however, that **gmXtal** expects the ions to be part of the force field used. Also, if ligands are selected for processing, the user is expected to provide the structure and force field parameters of the ligand, including hydrogens, because at this stage, **gmXtal** cannot automatically parameterise or add hydrogen atoms to ligands.

The mmCIF files are parsed with the BIO.PDB [29] and PDBeCIF [30] packages. The cleaning of the input structure is performed with standard routines from the Bio.PDB [29] and MDAnalysis [31, 32] packages. If needed, the structure is fixed either with pdbfixer [33], or with Modeller [34]. The protonation states of titratable residues can either be decided upon by the user, or automatically with *pdb2gmx*, based on pKa predictions by PROPKA [22, 35] or pKa-ANI [36].

Crystals are reconstructed using routines from the GEMMI library [37], which generates symmetry matrices from the symmetry information in the structural files available in PDBx/mmCIF format under *_symmetry:space_group_name_H-M entry*. GEMMI generates all symmetry matrices that correspond to the particular symmetry group. The generated matrices are so-called Seitz matrices $\{\textbf{R} | \textbf{t}\}$, which are transformation matrices in the cell coordinate space and thus, have to be translated into Cartesian space, $\textbf{M}$. This is achieved by applying the following transformations:

1 $$\begin{aligned} \textbf{M} = \textbf{A} \cdot \textbf{R} \cdot \textbf{A}^{-1} \end{aligned}$$

Here, $\textbf{R}$ is the rotational part of the Seitz matrix, and $\textbf{A}$ is the coordinate transformation matrix:

2 $$\begin{aligned} \textbf{A} = \begin{pmatrix} a &{} b \cos (\gamma ) &{} c \cos (\beta ) \\ 0 &{} b \sin (\gamma ) &{} -c \sin (\beta ) \cos (\alpha ^*) \\ 0 &{} 0 &{} c \sin (\beta ) \sin (\alpha ^*) \end{pmatrix} \end{aligned}$$

with *a*, *b*, *c* the lengths of the unit cell vectors, and $\alpha$, $\beta$, and $\gamma$ the angles between these vectors. Together these six parameters determine the geometry of the unit cell. The parameter $\alpha ^*$ is defined as

3 $$\begin{aligned} \cos (\alpha ^*) = \frac{\cos (\beta ) \cos (\gamma ) - \cos (\alpha )}{\sin (\beta ) \sin (\gamma )} \end{aligned}$$

The translation vectors are computed from the translation part $\textbf{t}$ of the Seitz matrices as follows:

4 $$\begin{aligned} \textbf{V} = \textbf{A} \cdot \textbf{t} \end{aligned}$$

With the matrices $\textbf{M}$ and translation vectors $\textbf{V}$, all crystallographic copies of asymmetric unit are created and then merged into a complete unit cell using routines from MDAnalysis [31, 32]. After the crystal structure is prepared, **gmXtal** writes a bash script for the GROMACS workflow.

#### GROMACS Workflow

Simulations of crystals are simulations of crystallographic unit cell of proteins. **gmXtal** prepares files for simulations of such system with GROMACS. By default, all simulations are performed with periodic boundary conditions, with the initial box dimensions set to experimental crystallographic unit cell parameters. The preparation module generates the symmetry mates of proteins, DNA chains and cofactors. Water molecules and ions, which are not visible in the experimental structure, still have to be added. After the addition of water and ions, the system has to relax and equilibrate to the desired temperature and pressure. All these steps are performed in GROMACS workflow. The GROMACS workflow includes the following steps: (i) adding hydrogens to the biomolecule with *gmx pdb2gmx* tool; (ii) adding water (solvent) molecules to the system; (iii) adding ions; (iv) running energy minimization; (v) running NVT and NPT equilibration; (vi) and performing a production run. The parameters for the energy minimization, equilibration, and production runs are provided as a part of **gmXtal** package, but the user can also provide own GROMACS parameter files (i.e., **.mdp**) for each of these steps.

Although not all solvent molecules are resolved in a crystal structure, these molecules determine density of the biomolecules in the crystal. Therefore, it is imperative that these missing solvent molecules are added to the unitcell. To determine the number of solvent molecules required to maintain the correct density, and hence unit cell volume, **gmXtal** creates multiple simulation boxes with different numbers of solvent molecules and checks which number preserves the unit cell volume during MD simulations. The number of solvent molecules is controlled with the **-scale** option of the *gmx solvate* routine. While the default value of 0.57 is suitable for creating simulation boxes of solvated proteins with the correct water density, this value may not be suitable for proteins in crystals (see results). Therefore, **gmXtal** probes several values of **-scale** parameter around 0.57 and recommends the optimal value based on an analysis of the MD simulations performed by the check module.

#### Check Module

The check module of **gmXtal** analyses the time-evolution of the unit cell volume in the production runs of systems with different numbers of added solvent molecules. For each frame the volume is computed as

5 $$\begin{aligned} V = a \times b \times c \times \sqrt{1 - \cos (\alpha )^2 - \cos (\beta )^2 - \cos (\gamma )^2 + 2\cos (\alpha )\cos (\beta )\cos (\gamma )} \end{aligned}$$

If the average unit cell volume in a production run is within $0.5\%$ of the experimental value, the system is considered optimal and **gmXtal** will recommend the user to proceed with the corresponding parameters. If the average volume is too far from the experimental volume in all trajectories, **gmXtal** will propose to rerun the whole workflow with a different set of **-scale** parameters for the *gmx solvate* routine. In this case **gmXtal** will output a file with the suggested new input parameters, for the preparation module.

#### gmXtal Input

The input required to run **gmXtal** in both preparation and check modes can either be provided interactively via the user interface, or read in from a file in **.yaml** format. If the input is provided interactively via the user-interface, **gmXtal** will save the input parameters locally in **.yaml** format, which can be reused or modified if needed. Examples of **.yaml** files are provided alongside with the script at https://gitlab.com/pbuslaev/gmxtal.

### Simulation Setup

**Fig. 2:**
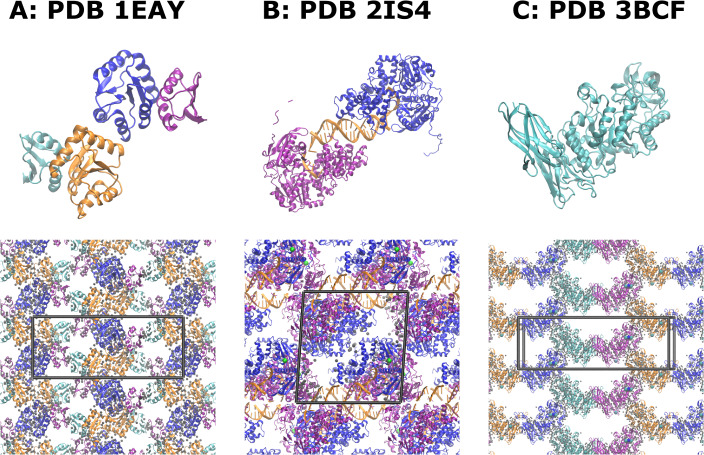
Structures of simulated systems (top row) and crystals build by gmxtal (bottom row). Different chains are shown in different colors. The unit cell is indicated by the black box. Structural waters are shown as gray spheres, ions as colored spheres

To validate the workflow implemented in **gmXtal** we set up crystals for MD simulations of (i) chey-binding (P2) domain of chea in complex with chey from *Escherichia coli* (PDB ID: 1EAY) [38], of (ii) UvrD-DNA-ADPNP ternary complex (PDB ID: 2IS4) [39], and of (iii) alpha-amylase B from *Halothermothrix orenii* (PDB ID: 3BCF) [40]. The 1EAY structure is a hetero-dimer, has a symmetry $P_{{2_1}{2_1}{2_1}}$, and has two copies of the protein dimer in the asymmetric unit. It also includes 124 structural water molecules. The 2IS4 structure is a homo-dimer that includes protein and DNA, has a symmetry $P_{1{2_1}1}$, and also includes two magnesium structural ions and 118 structural water molecules. The 3BCF structure is a monomer with $C_{121}$ symmetry. The structure contains also four calcium and one sodium structural ions, as well as 256 crystallographic water molecules. The structures of the initial complexes as well as the fully solvated crystals generated by **gmXtal** are shown in Fig. 2.

After the construction of the crystals, these systems were simulated with GROMACS2022 [25, 26] using periodic boundary conditions. The Amber ff99SB-ildn force field was used to model the interactions between the atoms [41]. The parameters used in our simulations are standard parameters recommended for using the AMBER family force fields with the GROMACS software [42]. Water was modeled with the TIP3P water model [43]. To all systems Na^+^ and Cl^-^ ions were added at 0.15 M concentration to neutralize the simulation box. Coulomb interactions were treated with the smooth PME method using a real-space cut-off of 1.0 nm and a grid spacing of 0.16 nm [44, 45]. Van der Waals interactions were modeled with a Lennard–Jones potential that was truncated at 1 nm and shifted using the Verlet modifier. The temperature was kept constant at 300 K with the v-rescale thermostat [46] using a time constant 0.5 ps^-1^. The pressure was kept constant at 1 bar with c-rescale barostat [47] using relaxation time of 5.0 ps. Trajectories were calculated using a leap-frog algorithm with a time step of 2 fs. The LINCS algorithm was used to constrain the lengths of bonds involving hydrogens in the protein and DNA [48, 49], while SETTLE was used to constrain the internal degrees of freedom of the water molecules [50]. All production runs were simulated for 20 ns. Prior to the production runs, the energy of all systems was minimized with the steepest descent method, first followed by a 100 ps simulation at constant temperature and volume, and then by a 100 ps simulation at constant temperature and pressure. All parameter files used for these simulations are provided as a part of **gmXtal** at https://gitlab.com/pbuslaev/gmxtal.

## Results

We performed simulations of three systems that have different symmetries and content of the asymmetric unit (Fig. 2). These systems were selected to demonstrate that **gmXtal** is applicable to a wide range of biomolecular complexes and crystallographic symmetries. Thus, we selected (i) a hetero-dimer and (ii) a protein-DNA complex with two copies of the asymmetric unit in the unit cell and (iii) a large enzyme with four copies of the asymmetric unit in the unit cell. Figure 2 shows the structural organization of the crystals that were build by **gmXtal**. The differences in crystallographic symmetries lead to differences in the empty spaces between the proteins, which **gmXtal** fills up with water during the preparation phase.

**Fig. 3:**
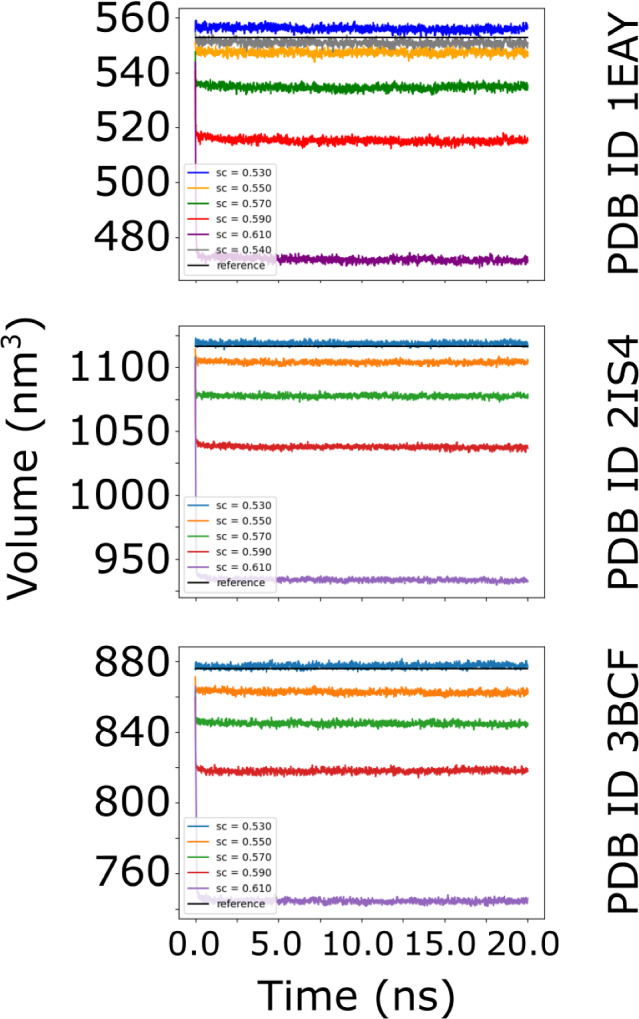
Time-evolution of the unit cell volume in simulations with varying numbers of water molecules added to the system. The water molecules were added with *gmx solvate* using different values for the **-scale** option: 0.53 — blue curve, 0.55 — orange curve, 0.57 — green curve, 0.59 — red curve, and 0.61 — purple curve. For 2IS4 and 3BCF the optimal **-scale** parameter is 0.53. For 1EAY system 0.53 is quite close to preserving the volume, but **gmXtal** nevertheless suggested running a second iteration with **-scale** values in-between 0.53 and 0.55. After the second iteration, **gmXtal** recommends **-scale** parameter to be set to 0.54 for 1EAY system. The volume evolution of 1EAY at 0.54 value of **-scale** parameter is shown in the top panel with gray color (Color figure online)

To determine the correct number of solvent molecules, we ran **gmXtal** with five different values of the **-scale** option of the *gmx solvate* program: 0.53, 0.55, 0.57, 0.59, and 0.61. These values determine the initial water density in the crystals. For each system a 20 ns MD trajectory was computed and the time-evolution of the volume was analyzed (Fig. 3).

For systems 2IS4 and 3BCF the experimental volume was reproduced with the **-scale** option set to 0.53. For 1EAY system, the experimental volume was not reproduced fairly well for any of the selected densities of added solvent. Instead, **gmXtal** recommended to prepare new systems with the **-scale** option in the range from 0.53 to 0.55, and run a new set of simulations. After this second iteration, we found that the optimal water density was achieved with the **-scale** option set to 0.54. The three examples suggest that, while the number of added solvent molecules cannot be predicted in advance, **gmXtal** provides a means to estimate that number. For crystals the optimal value for the **-scale** option of *gmx solvate* tool can be different from the recommended value for solvating proteins in water. The crystals created by **gmXtal** provide the starting point for investigating the properties of biomolecular crystals by means of MD simulations, just as Herman Berendsen et al. did, almost four decades ago [6].

## Conclusion

To summarize, we have presented **gmXtal**, a python-based toolbox for automatically generating hydrated crystals from protein data bank files. We believe that this tool can be useful for performing simulations of crystals as it avoids the tedious procedure of setting up crystal models manually and in particular determining the number of solvent molecules required to maintain the experimental density and unit cell volume. We expect that our tool will pave the way to systematically explore the effects of the crystal environment on the properties of biomolecular systems, including catalytic activity and p$K_\text {a}$s of titratable amino acids, which can be dramatically different between solution and crystal, as for instance in PYP, for which it was suggested that an Arginine is deprotonated in the crystal [51].
